# Help-seeking behaviors and determinant factors among women exposed to intimate partner violence in East Africa based on recent demographic and health survey data: a multilevel analysis

**DOI:** 10.3389/fpsyt.2024.1402704

**Published:** 2024-09-26

**Authors:** Mamaru Melkam, Angwach Abrham Asnake, Yohannes Mekuria Negussie, Meklit Melaku Bezie, Zufan Alamrie Asmare, Hiwot Altaye Asebe, Beminate Lemma Seifu, Bezawit Melak Fente

**Affiliations:** ^1^ Department of Psychiatry, College of Medicine and Health Science, University of Gondar, Gondar, Ethiopia; ^2^ Department of Epidemiology and Biostatistics, School of Public Health, College of Medicine and Health Sciences, Wolaita Sodo University, Wolaita Sodo, Ethiopia; ^3^ Department of Medicine, Adama General Hospital and Medical College, Adama, Ethiopia; ^4^ Department of Public Health Officer, Institute of Public Health, College of Medicine and Health Sciences, University of Gondar, Gondar, Ethiopia; ^5^ Department of Ophthalmology, School of Medicine and Health Science, Debre Tabor University, Debre Tabor, Ethiopia; ^6^ Department of Public Health, College of Medicine and Health Sciences, Samara University, Samara, Ethiopia; ^7^ Department of General Midwifery, School of Midwifery, College of Medicine and Health Sciences, University of Gondar, Gondar, Ethiopia

**Keywords:** multilevel analysis, help seeking, behavior, intimate partner, violence

## Abstract

**Introduction:**

Human rights violations and violence against women are serious public health issues that have numerous detrimental repercussions on one’s physical, emotional, sexual, and reproductive health. According to studies, women’s perceptions and traits of violence are highly predictive of their likelihood of seeking help against violence. Even though intimate partner violence is a huge challenge nowadays in Africa, there is a low level of help-seeking behavior. Conducting this study at the East African level on help-seeking behavior can provide a clue for policy-makers. Therefore, this study aimed to reveal the prevalence of help-seeking behavior against intimate partner violence and determinant factors among women in East Africa.

**Method:**

Multilevel logistic regression analysis was carried out among East Africans using recent demographic and health survey data. A total of 7,387 participants aged 15 to 49 years were included in this study from East African countries. Individual- and community-level variables were considered to determine the associated factors with help-seeking behaviors against intimate partner violence with 95% CI and AOR.

**Results:**

The prevalence of help-seeking behavior against intimate partner violence among women was 38.07% with 95% CI (36.96%, 39.18%). Husbands drink alcohol [AOR = 1.46: 95% CI (1.33, 1.61)], women who have work [AOR = 1.33: 95% CI (1.19, 1.50)], and women with higher educational status [AOR = 1.36: 95% CI (1.16, 1.59)] were factors associated with help-seeking behavior against intimate partner violence.

**Conclusion:**

Approximately four out of 10 women were seeking help for intimate partner violence in East Africa. Husbands drinking alcohol, women’s high educational status, and women having occupations were the factors that were associated with help-seeking behaviors against intimate partner violence.

## Introduction

Intimate partner violence (IPV) is a severe public health issue that includes physical and/or sexual abuse, stalking, and emotional hostility committed by a former or present intimate partner beyond cultural boundaries (1). The international society is beginning to acknowledge the physical abuse of women as a grave human rights violation. According to scholars, women in underdeveloped countries are more likely than women in developed countries to be physically abused by intimate partners (2, 3). Investigations show that between 15% and 71% of women who have ever been in a relationship have experienced physical or sexual abuse at the hands of an intimate partner (4). When faced with IPV, women may decide to seek assistance from both official and unauthorized sources. Traditional sources of support include non-governmental organizations, shelters, and other services connected to intimate partner violence as well as medical or legal professionals, clergy, and local leaders (5). In addition to IPV’s significant global incidence, women who have experienced IPV report a low prevalence of help-seeking behavior (1, 6). Research demonstrates a distinct pattern of how women’s behavior in seeking help is influenced by gender parity in a culture. Women in low-income nations with high levels of gender inequality and strict gender norms are less likely to ask for help than women in nations with higher levels of gender equality and more flexible gender roles (7, 8). Some women believe that the abuse is not serious enough to warrant seeking help, while others, mostly as a result of cultural norms, fail to identify IPV or accept it as the norm (9). Compared to women who are assaulted emotionally or sexually, those who experience physical abuse are more likely to seek assistance (10).

According to the WHO’s multi-country research on intimate partners, 55%–95% of women who had experienced physical or sexual abuse never sought assistance from a formal institution (11). IPV is a significant public health issue in the United States, where 36% of women would, at some point in their lives, encounter rape, physical abuse, or stalking at the hands of an intimate partner (12). There has been a variation in the prevalence of intimate partner violence both nationally and locally, which evidences signify low levels of communal empowerment for women (13, 14). Furthermore, women who experienced violence both before and throughout their pregnancies reported experiencing higher levels of intensity and frequency of pregnancy-related violence (15). However, there is a huge burden of intimate partner violence in low- and middle-income countries, there are limited help-seeking behaviors, as shown in studies in Sub-Saharan African countries at 19.75% (16). The prevalence of IPV ranges from 26.7% to 39.3% in East Africa, and 15.6% to 19.0% of women said they have experienced all three types of IPV at the same time (17). The prevalence of IPV was also from 10.2% to 20.0% and 30% among women in Rwanda and Ethiopia, respectively (18, 19). The burden of IPV accounts for 22.9% to 48.4% in Burundi and 27% to 61% in Tanzania (20, 21). The potential negative effects of intimate partner violence on health extending beyond the physical trauma that leads to emotional and severe mental conditions are well acknowledged and understood (22). Survivors of intimate partner violence typically turn to their relatives and close companions for support, a tactic known as “help-seeking from the informal structures” (9). Although there is growing evidence of the risk factors for intimate partner violence in low-resource countries, the benefits of seeking help after experiencing violence in African countries have received little attention (23). Some factors were associated with help-seeking behaviors of survivors of IPV in different studies. Wealth index, mass media exposure, place of residence, education level, women’s autonomy in household decision-making, partner’s controlling behavior, current employment status, partner’s education, and partner’s alcohol use were associated with help-seeking survivors of intimate partner violence.

Examining the variables associated with IPV victims in East Africa help-seeking could help guide secondary prevention initiatives meant to increase IPV disclosure to official and unofficial sources. The prevalence of intimate partner violence in East Africa was 37.14%; no study shows their help-seeking (24). Despite the huge impact of intimate partner violence, the help-seeking of the survivors was not studied well particularly in East Africa. Therefore, this study revealed help-seeking behaviors and determinant factors among women exposed to intimate partner violence in East Africa based on recent demographic and health survey (DHS) data.

## Methods

### Study area

A multilevel analysis was conducted using recent demographic health survey data of East African countries: Burundi (2,122), Ethiopia (3,142), Comoros (390), Malawi (10,249), Rwanda (4,601), Uganda (4,213), Zambia (4,160), and Zimbabwe (4,300). The wide range of objectives includes indicators of fertility, reproductive health, maternal and child health, mortality, nutrition, and self-reported health on self-reported data from East African countries’ DHS. The datasets of the East African DHS included men, women, children, births, and households’ data. The individual record dataset (IR file) was the data that was used for this study extracted from this survey. A total of 816 clusters were used in the equal probability selection procedure from the household health survey framework. The study participants including women from East African countries between the ages of 15 and 49 who had intimate partner violence served as the source population. A total of 7,387 weighted sample size of eight East African countries’ DHS was determined for this secondary data analysis with women who had intimate partner violence, with 816 enumeration areas (EAs) or clusters. The detailed data can be accessed comprehensively by clicking on the official link (http://www.dhsprogram.com/) (25).

### Variables of the study

#### Dependent variables

The outcome variable was the behavior related to seeking help for IPV. Only women who had ever experienced intimate partner violence were asked if they had ever sought assistance from anyone. The common sources for help were their own family, husband’s/intimate partner’s family, friends, and chief/other national government administrative officers. The query to screen help sought was “sought help from someone” with a yes/no response. Participants who responded “yes” were recoded “1” and participants who responded “no” to the question were recoded as “0” (16, 26).

#### Independent variables

Independent variables were extracted from the East African countries’ recent DHS, including household variables, wealth index, and reproductive-related variables. The extracted independent variables incorporated in this study were sex of the household head, age, distance from the health facility, number of children, current marital status, ethnicity, religion, education level of the respondent and partner, occupation of the respondent, the partner age difference between spouse, residence, and husband’s/partner’s alcohol use, which were used as individual-level variables. The community-level variables used for this study included place of residency (urban and rural), educational level (low and high), wealth index (low and high), and media exposure (low and high). The distribution of the proportion values that were computed for each community-level variable was examined using a histogram. Lastly, for dichotomously skewed and normally distributed variables, median and mean values were employed, respectively.

### Data management and analysis

Stata version 14 was used for data extraction, coding, cleaning, and analysis. Among the descriptive data filled in a table and text were frequency and percentage. The non-proportionate allocation of the analysis and the sample’s representativeness were done using sample weight with cluster. To maintain the hierarchical structure of the gathered data, a mixed multilevel analysis was done. To identify the associated variables to be included in the multivariable analysis with a *p*-value less than 0.25, a multilevel bi-variable logistic regression analysis was performed. Multilevel multivariable logistic regression analysis was used to identify the statistically significantly linked variables with *p*-values less than 0.05. An adjusted odd ratio (AOR) with 95% confidence interval (CI) was then calculated.

The multivariable multilevel logistic regression study involved four models of analyses. No explanatory variables were used in the first model, sometimes referred to as the null model. In the second model, community-level variables were fitted only; in the third model, community-level variables were fitted; and in the fourth model, both individual- and community-level variables were fitted. The models were compared, and the Akaike Information Criterion (AIC) and deviance were used to assess each model’s fitness; the model with the lowest score was deemed to be the best fit. The degree of heterogeneity among intimate relationship violence among the clusters was also assessed by intra-class correlation $\left(\text{ICC}\right)=\frac{VA}{VA+3.29}*100\%$ . The portion of each individual’s reported intimate partner violence variance can be attributed to variations among clusters. To measure the variation in intimate partner violence among clusters, median odds ratio (MOR) =e^0.95√VA^ was employed (27). The degree of homogeneity and the measurement of the odd ratio scale variation of intimate partner violence among the clusters were carried out. In the end, variables with a *p*-value less than 0.05 that were statistically significantly linked to intimate partner violence were found, and the AOR with 95% confidence interval was calculated. Variable inflation factor (VIF) was conducted to check for multicollinearity, and the result is less than 10, which indicates no multicollinearity in this study.

## Results

### Descriptive characteristics of respondents

A total of 7,387 study participants who had intimate partner violence and were between the ages of 15 and 49 were included in this secondary data analysis of East African DHS. Among the study participants, 24.34% were between the ages of 25 and 29, and 52.55% were women who had completed primary education. Among women who had intimate partner violence, 59.88% were Christians. Among the study participants, 75.21% had occupations, and 80.05% had partners with occupations. Among the participants, 62.46% had a problem with health facility distance, and 46.01% had partners who had drunk alcohol. Among the study participants, 71.41% were from rural areas (**Table 1**).

**Table 1:** Descriptive characteristics of East African countries’ study participants for help-seeking behavior against IPV (*N* = 7,387).

Variables	Category	Weighted frequency (*n* = 17.7)	Percentage (%)
**Age**	15–24 25–29 30–34 35–39 40–44 45–49	1,782 1,798 1,682 1,119 704 302	24.12 24.34 22.77 15.15 9.53 4.09
**Mother’s education**	No education Primary Secondary Higher	1,022 3,882 2,216 267	13.84 52.55 30.00 3.61
**Partner’s educational level**	No education Primary Secondary Higher	1,744 2,832 2,258 553	23.61 38.34 30.57 7.49
**Religion**	Christian Muslim Other religions^a^	4,423 618 2,346	59.88 8.37 31.76
**Partner’s occupation**	Not working Working	1,474 5,913	19.95 80.05
**Mother’s occupation**	Not working Working	1,831 5,556	24.79 75.21
**Partner’s alcohol use**	Yes No	3,399 3,988	46.01 53.99
**Distance of health facility**	Yes No	2,773 4,614	37.54 62.46
**Media exposure**	Yes No	4,992 2,395	67.58 32.42
**Economic status**	Poorest Poorer Middle Richer Richest	1,465 1,539 1,405 1,561 1,417	19.83 20.83 19.02 21.13 19.18
**Number of children**	0 1–2 3–4 5	1,244 5,730 401 12	16.84 77.57 5.43 0.16
**Current marital status**	Married Separated Widowed/divorced	6,321 203 863	85.57 2.75 11.68
**Community-level wealth index**	High Low	392 424	48.04 51.96
**Community-level media exposure**	High Low	485 331	59.44 40.56
**Community-level education**	High Low	423 393	51.84 48.16
**Residence**	Urban Rural	2,112 5,275	28.59 71.41

a Other religions include Orthodox and atheist.

### Prevalence of help-seeking behaviors among IPV

The prevalence of help-seeking behavior against violence among women who had intimate partner violence was 38.07 with 95% CI (36.96%, 39.18%).

### Model fitness and statistical analysis

Not seeking help against violence within the cluster was associated with 2.53 of the respondents’ changes in the ICC of the null model (model I). The MOR of intimate partner violence in the null model was 2.53, indicating that there was variation between the clusters. The odds of individuals experiencing intimate partner violence were 1.73 times higher in the cluster with a higher risk of these disorders than in the cluster with a lower risk, assuming that a single participant was randomly picked from each of the two clusters. Model IV was the well-fitting model for this investigation since it had the lowest AIC and deviation value (**Table 2**).

**Table 2:** Multilevel multivariable logistic regression analysis of East African countries’ DHS (n = 7,387).

Variable	Null model	Model I	Model II	Model III
Age				
15–24 25–29 30–34 35–39 40–44 45–49		1 0.99 (0.64, 1.55) 0.94 (0.61, 1.47) 1.42 (0.85, 2.34) 1.40 (0.79, 2.49) 0.93 (0.41, 2.08)		1 1.06 (0.92, 1.22) 1.11 (0.96, 1.28) 1.09 (0.93, 1.28) 1.25 (0.13, 1.50) 1.17 (0.91, 1.52)
Wealth index				
Poorest Poorer Medium Richer Richest		1 0.95 (0.58, 1.54) 0.63 (0.37, 1.06) 0.95 (0.26, 1.78) 0.75 (0.40, 1.40)		1 1.17 (0.96, 1.41) 1.03 (0.85, 1.24) 1.04 (0.86,1.24) 0.95 (0.81, 1.12)
Partner’s education				
No education Primary Secondary Higher		1 1.33 (0.80, 2.23) 1.42 (0.82, 2.47) 0.77 (0.36, 1.65)		1 1.06 (0.91, 1.24) 1.05 (0.89, 1.25) 0.87 (0.68, 1.11)
Partner’s alcohol use				
Yes No		1.71 (1.25, 2.33) 1		1.46 (1.33, 1.61) 1
Mother’s occupation				
Working Not working		1.38 (0.96, 1.98) 1		1.33 (1.19, 1.50) 1
Mother’s education				
No education Primary Secondary Higher		1 0.53 (0.32, 0.89) 0.75 (0.52, 1.09) 1.48 (0.90, 2.44)		1 0.87 (0.66, 1. 89) 0.95 (0.84, 1.07) 1.36 (1.16, 1.59)
Partner’s occupation Working Not working		0.52 (0.21, 1.27) 1		0.99 (0.74, 1.33) 1
Community-level variables				
Residence Urban Rural			1 1.04 (0.77, 1.39)	1 1.53 (0.44, 1.12)
Media exposure				
High Low			1 1.12 (0.81,41.55)	1 0.88 (0.63,1.23)
Education				
High Low			1 1.01 (0.75, 1.37)	1 0.99 (0.72, 1.35)
Economic status				
High Low			1 0.88 (0.64, 1.21)	1 1.10 (0.79, 1.53)
**Likelihood ratio**	-4,907.5802	-4,832.5424	-553.83734	-508.07381
**ICC**	0.00478	0.00812	0.00678	0.0076
**Deviance**	9,815.1603	9,665.0848	1,066.9109	1,016.1476
**AIC**	9,819.16	9,705.106	1,076.911	1,062.148
**BIC**	9,832.975	9,843.255	1,100.433	1,170.349
**MOR**	2.53			

ICC, intra-class correlation; MOR, median odds ratio; AIC, Akaike Information Criterion; BIC, Bayesian Information Criteria.

### Associated factors of help-seeking behavior

In the multilevel bi-variable analysis, factors associated with help-seeking behavior were the age of the respondent, women’s education, partner’s education, partner’s alcohol use, women’s occupation, partner’s occupation, and wealth index at the individual level with a *p*-value less than 0.25. In the multilevel multivariable analysis, partner’s alcohol use, higher educational level of women, and women who have occupations were significantly associated with help-seeking behavior for their violence with a *p*-value less than 0.05. The odds of having help-seeking behavior for their violence among study participants who have husbands who drink alcohol was 1.46 times higher compared with the study participants whose husbands did not drink alcohol [AOR = 1.46: 95% CI (1.33, 1.61)]. The odds of seeking help for their violence were 1.33 times higher among women who have work than other subjects who have no work [AOR = 1.33: 95% CI (1.19, 1.50)]. Study participants who have higher educational status were 1.36 times higher compared with study participants who have no education [AOR = 1.36: 95% CI (1.16, 1.59)] (**Table 2**).

## Discussion

The main purpose of the study was to look into community- and individual-level variables of help-seeking behavior against intimate partner violence among women aged 15 to 49. The prevalence of help-seeking behavior against violence among women who had intimate partner violence was 38.07% with 95% CI (36.96%, 39.18%). This finding is in line with other studies conducted in Sub-Saharan African countries at 38.77% (16).

This finding is lower than other studies conducted in India at 48.8% (28). The possible reason for this discrepancy might be due to the difference in sample size; the Indian study had a small sample, but this study combined data from different countries with a high sample size (28). In other words, this finding is higher than other studies conducted in Afghanistan at 20% (23) and in developing nations at 34.88% (26). The probable evidence for this discrepancy could be the effect of low-level intimate partner violence, which implies that study participants who sought help were lower in Afghan than in East African countries (23).

Related to factors, husband drinking alcohol is one of the associated factors with help-seeking behaviors. This association is consistent with other studies conducted in Ghana (29), Ethiopia (30), and Africa (31). This association could be because of the effect of consuming alcohol; the user may act violently and make other people less willing to discuss a peaceful solution to the conflict in the relationship, though women sought help against violence (31). Moreover, binge drinking can cause other household pressures like financial hardships, children issues, and marital challenges, which enable women to seek help to solve those problems against violence (29). This demonstrated the way education might encourage female opinions that promote victim safety and personal relevance to make the correct decisions by helping them grasp what is correct about IPV (30).

The other factor which was associated with help-seeking behavior was women’s higher educational status. This association is in concordance with other studies conducted in Ethiopia (32), India (33), and Sub-Saharan Africa (34). The probable reason for this association could be the effect of lack of knowledge of mitigating factors, restricted access to legal counsel, and a rise in the acceptance of detrimental conventional views for women (32). Furthermore, there is an opportunity that it is connected with other elements like power to make decisions and resources (35). People can learn about the ethical foundations of society through education, which can also expose them to international debates that oppose IPV and promote help-seeking behavior (34). According to health behavior theory, education is a powerful tool for changing behavior through raising awareness and knowledge (36). This may be because better-educated women in Africa are expected to follow the social norms, which may cause them to not keep quiet about IPV. Additionally, educated women receiving violence are expected to pass the challenges including mental or psychological barriers like shame and anxiety (33). This could be a result of literate women having greater access to knowledge and information, which may modify and influence women’s views and help them understand what is and is not acceptable.

Another factor associated with help-seeking behavior against intimate partner violence was women who had an occupation. This association is in line with other studies conducted in Ethiopia (30) and Niger (37). The possible reason for this association might be the effect of having their financial support to process the help sought (30). Women’s perspectives about equality in a relationship, as opposed to allowing violence, may be influenced by empowerment, which may also help them feel more confident in their ability to defend what is acceptable to them (37). The other possible reason might be the effect of having an occupation that creates a feeling of independence to protect their human right against intimate partner violence.

All women are supposed to seek help for intimate partner violence practically. To practically lessen or remove obstacles to help-seeking, this research indicates their behavior to mitigate the negative effects of intimate partner violence on the quality of life and health outcomes of abused women. The fact that many of these women were unable to defend against their abusive situations, this study will motivate them to ask for their rights. This study also provides an entry for policymakers and women’s rights protection organizations to enhance help-seeking behavior to mitigate the burden of intimate partner violence.

## Limitations of the study

Even though this study had many strong sides, it has its weaknesses. One of the limitations of this study is that the cause-and-effect relationship of factors with outcome variables was not provided due to the cross-sectional nature of this study. The other limitation could be the social desirability of the study participants to report their partners’ violence.

## Conclusion and recommendation

Nearly four out of 10 participants were seeking help for intimate partner violence in East Africa. Husbands drinking alcohol, women’s high educational status, and women having occupations were the factors that were associated with help-seeking behaviors against intimate partner violence. Enhancing the level of education and women’s employment in different jobs is crucial for them to raise a response of help-seeking for IPV. This finding can be used as a clue for policymakers and other stakeholders who need to raise the behavior of help-seeking. Future researchers are recommended to conduct advanced methods to predict the exact barriers of help-seeking behavior.

## Data Availability

The datasets presented in this study can be found in online repositories. The names of the repository/repositories and accession number(s) can be found in the article/Supplementary Material.
